# Advanced glycation end products (AGEs) estimated by skin autofluorescence are related with cardiovascular risk in renal transplant

**DOI:** 10.1371/journal.pone.0201118

**Published:** 2018-08-01

**Authors:** Jesus Calviño, Secundino Cigarran, Lourdes Gonzalez-Tabares, Nicolas Menendez, Juan Latorre, Sonia Cillero, Beatriz Millan, Carmen Cobelo, Ana Sanjurjo-Amado, Jansen Quispe, Alba Garcia-Enriquez, Juan J. Carrero

**Affiliations:** 1 Nephrology, Hospital Lucus Augusti, Lugo, Spain; 2 Nephrology, Hospital da Costa, Lugo, Spain; 3 Department of Medical Epidemiology and Biostatistics (MEB), Karolinska Institutet, Stockholm, Sweden; Hospital Universitario de la Princesa, SPAIN

## Abstract

**Background:**

Advanced glycation end products (AGEs) accumulation, a measure of cumulative metabolic stress, constitute a novel pathogenic mechanism involved in aging, diabetes, cardiovascular (CVD) and chronic kidney disease (CKD). Despite removal of uremic toxins and AGEs after a successful renal transplant (RT), CVD remains the leading cause of mortality. We hypothesized that AGEs measurement by Skin Autofluorescence (SAF) might be useful even after a successful RT and thus reflect the high cardiovascular risk burden of these patients.

**Methods:**

189 stable RT (61% men, aged 56±13.0 years), CKD stages 1–4 and >12 months since RT were enrolled. Variables collected comprised comorbid history, medication use, smoking habit, routine biochemistry, subclinical atheromatosis by ankle-brachial-index (ABI) and allograft resistivity index (RI), 24-h ABPM, anthropometry and handgrip strength. AGEs were measured by SAF and expressed in arbitrary units (AU). Vascular age was estimated by Koetsier´s formula (SAF-0.83/0.024) and expected 10-years cardiovascular death risk was calculated with the REGICOR score.

**Results:**

Mean SAF was 3.00±0.83 AU and estimated vascular age 90±34.7 years (30 years above biological age). SAF was higher among men (3.10±0.91 vs 2.81±0.66), diabetic nephropathy (3.49±0.75 vs 2.96±0.83) and steroid users (3.14±0.86 vs 2.71±0.69). We observed a positive correlation of SAF with night-systolic blood pressure (r = 0.25, p = 0.001), parathormone (r = 0.20, p<0.01), phosphate (r = 0.28, p<0.001) and negative with hemoglobin (r = -0.29, p<0.001), CKD-EPI (r = -0.32, p<0.001), albumin (r = -0.17, p<0.05), and dynamometry (r = -0.20, p<0.01). Subclinical vascular atheromatosis (ABI and RI) as well as the REGICOR scale (r = 0.35 p<0.001) were also correlated with SAF. In multivariable analysis age, gender, steroid use, serum phosphate and handgrip strength remained independently associated with SAF.

**Conclusions:**

SAF levels are elevated in RT patients and correlate with CVD risk. Besides age and male sex, our results suggest that phosphate overload, steroid use and nutritional status are important factors linking to AGEs accumulation.

## Introduction

Advanced glycation end products (AGEs) are a variety of compounds produced by the nonenzymatic Maillard reaction that binds reducing sugars with the free amino groups in proteins, lipids or nucleic acids [1]. AGEs can be degraded by enzymes, such as glyoxalase (I and II), and they can also be modified by innate defense machineries, such as lysozyme, which sequesters AGEs and accelerates their renal excretion [2]. AGEs receptor 1 (AGER1) binds AGEs, leading to their sequestration and detoxification, resulting in antioxidant properties. AGE–specific receptor (RAGE) is a multi-ligand transmembrane receptor that binds many ligands, including AGEs, triggering inflammatory and oxidative injury. A misbalance between RAGE and AGER1 levels result in suppression of the antioxidant defense system and increased levels of pro-oxidant mechanisms [2]. Accumulation of AGEs has been proposed to be a pathogenic mechanism of oxidative stress, inflammation and structural tissue damage leading to chronic vascular complications in different disorders including aging, diabetes and chronic renal disease (CKD) [3, 4]. Diabetic nephropathy illustrates a classical model for AGEs pathogenic injury [5]. More recently, it has been suggested that AGEs accumulation may be involved in the progression of CKD and cardiovascular disease (CVD) both in diabetic and in non-diabetic patients [6,7].

Levels of plasma or urine AGEs have been linked with the severity and prognosis of diabetes, renal and CVD. However, these samples frequently fail to reproduce tissue levels, which more precisely reflect long-standing negative interactions of AGEs [8, 9]. Whereas direct determination of tissue AGEs is an invasive and expensive procedure, skin autofluorescence (SAF), a noninvasive automated measure of tissue AGEs accumulation has recently become an easy and validated method for clinical use. SAF has been shown to be highly correlated with serum AGEs concentrations and more importantly reflects long-standing AGEs accumulation that is associated with tissue and end organ damage [10,11]. Several reports have shown SAF to be independently associated with aging and the development of long-term complications of diabetes, CKD progression and CVD mortality [11–16].

In CKD, accumulation of AGEs is attributed to impaired renal clearance, increased endogenous formation and excess dietary intake [17, 18]. Renal transplantation (RT) is the treatment of choice for end stage renal disease. Despite conferring maximal kidney function and survival benefits, postransplant cardiovascular risk remains high even with a healthy graft [19]. Although plasmatic clearance of AGEs dramatically improves after RT, its removal from slow-turnover tissues might be difficult to achieve and may contribute to this high CVD burden [8, 20]. Some studies have linked tissue levels of AGEs, estimated by SAF, with both chronic allograft dysfunction and a poorer CVD outcome [21, 22]. However, the magnitude of AGE contribution to the important cardiovascular risk of these patients remains to be clarified. Moreover, biochemical, nutritional and subclinical atherosclerotic markers related with AGE accumulation after renal transplantation have not been studied in depth.

Therefore, we hypothesized that AGEs measurement by Skin Autofluorescence might be useful to screen cardiovascular risk even after long-term successful renal grafting. For this aim, we assess SAF in stable RT, with well-characterized clinical and biochemical parameters, in order to investigate factors linked with tissue accumulation of AGEs and analyze its potential significance as a marker of recipient’s cardiovascular risk.

## Materials and methods

### Study population

A total of 189 stable renal transplant patients followed at the nephrology outpatient clinic (EOXI Lugo-Cervo-Monforte, Lugo-Spain) were asked to participate in this observational cross-sectional study and written informed consent was obtained from all subjects. Recruitment was performed between May 2016 to June 2017. All procedures were in accordance with the ethical standards for research involving human subjects in the Helsinki Declaration and were approved by the local ethics committee (Comité Autonómico de Ética da Investigación de Galicia. Dictamen del Comité Santiago-Lugo; code number 2017/236). Only Caucasian patients with stable renal function for the previous three months were included. Subjects under 18 years old, pregnant women, those with acute inflammatory or infectious disease, or those hospitalized in the previous 3 months were excluded. Patients with a double kidney-pancreas graft, type 1 diabetes mellitus or those with uncontrolled malignancy on chemotherapy were not included. As AGEs levels may improve after a renal graft, only those patients followed for more than 12 months after transplantation were screened. To minimize confounding that would be introduced by poor allograft function; participants with estimated glomerular filtration rate (eGFR) of less than 15 mL/min per 1.73 m^2^ were also excluded. Immunosuppressive therapy generally consisted of prednisone with a calcineurin inhibitor (mainly tacrolimus) or m-TOR inhibitor, combined with mycophenolate.

### Skin autofluorescence measurement (SAF)

SAF was assessed by a validated reader (Diagnoptics Technologies BV, Groningen, the Netherlands) as previously described in detail [10]. The autofluorescence reader (AFR) is a desk-top device based on the characteristic fluorescent properties of certain AGEs to quantify their level of accumulation in the skin. It illuminates a skin surface of 1 cm^2^, guarded against surrounding light, with an excitation light source between 300 and 420 nm (peak excitation approximately 370 nm). Only light from the skin is measured with a spectrometer in the 300–600 nm range, using a 200 mm glass fiber. SAF is calculated as the ratio between the emission light and reflected excitation light, multiplied by 100 and expressed in arbitrary units (AU). Reproducibility of the device has been tested in different study populations and showed a mean relative error in skin AF of ∼5% [10]. Mean age-corrected skin AF per intraindividual seasonal variance or even per AFR system did not differ significantly as well.

Measurements were performed at room temperature with the patient in a seated position, at the ventral side of the lower arm approximately 10–15 cm below the elbow fold. Care was taken to perform the measurements at normal skin site, that is, without visible dermatosis, bruising or pigmentation disorders, malignancies or other skin abnormalities including arteriovenous fistula. Autofluorescence was calculated by automated analysis and was therefore observer-independent. Using skin AF result we calculated “vascular age” by the relation previously described by Koetsier et al [23], for age and SAF in a healthy Caucasian population [vascular age = (skin AF– 0.83) / 0.024)]

### Clinical, laboratory and other parameters

History of cardiovascular disease: ischemic heart disease (coronary syndrome or revascularization), cerebrovascular and peripheral artery disease (amputation or revascularization) were extracted from medical records. In addition, peripheral artery disease (PAD) was also screened by means of the ankle-brachial index (ABI). A pathologic ABI was defined as a value ≤0.9 or ≥1.4. Subclinical atheromatosis was assessed by means of the the intrarenal resistivity index (RI) of the graft. RI, measured by doppler-ultrasound examination at three different places of the graft, was calculated as [1 − (Vmin ÷ Vmax)] where Vmax is the peak of systolic velocity and Vmin the minimal diastolic velocity. Results are expressed as the average of all these measures. A pathologic RI was defined as a value ≥ 0.8. Blood pressure was monitored by 24 h-ambulatory blood pressure monitoring (24h-ABPM). Concomitant medications including antihypertensive treatment, immunosuppression (IS) and statins were also recorded.

The REGICOR (REgistre GIroní del COR) formula was used to estimate the 10-year risk of mortality from cardiac disease. This is a modified Framingham Risk Equation based on age, gender, systolic and diastolic blood pressure, smoking status, diabetes, total cholesterol and high-density lipoprotein cholesterol that has been validated for the Spanish population [24].

Anthropometric measurements, including mid-upper arm circumference (MUAC), height, waist and weight were performed during the clinical examination. Body mass index (BMI) was calculated (kg/m^2^) and handgrip muscle strength measured in the non-dominant arm using the Baseline Hydraulic Hand Dynamometer (NexGen Ergonomics Inc, Quebec. Canada).

Blood and urine laboratory measurements (Cobas system, Roche diagnostics, Basel, Switzerland) included serum creatinine, uric acid, proteinuria (albumin to creatinine ratio), bicarbonate, calcium, phosphate, parathyroid hormone (PTH), vitamin D, C-reactive protein, ferritin, hemoglobin, glycated hemoglobin (HbA1c) and lipid profile. Albumin and transferrin concentrations were used as biochemical markers of nutritional status. All measurements were part of our routine patient visits and analyzed centrally at the hospital laboratory. eGFR expressed as mL/min per 1.73m^2^ was estimated using the Chronic Kidney Disease Epidemiology Collaboration equation (CKD-EPI) [25]. Phosphate tubular reabsorption rate was calculated as: [1- ((urine phosphate x serum creatinine) / (urine creatinine x serum phosphate)) x 100].

### Statistical analyses

Statistical analyses were performed using SPSS version 21 (SPSS Inc, Chicago, IL). The primary goal was to evaluate SAF (AU) relationship with clinical, biochemical, nutritional and surrogate CVD markers. Normal distribution of the variables was evaluated using the Kolmogorov-Smirnov test. Accordingly, both parametric and non-parametric tests were used as appropriate. Differences in continuous variables for important selected strata were tested using the t-student or Mann-Whitney *U*-test. All results were expressed as means and standard deviation unless otherwise stated. Pearson´s or Spearman´s correlation coefficient, as appropriate, was used to evaluate bivariate relationships between continuous variables. The χ2 test or Fisher exact test, if applicable, were used to compare categorical variables by cross tabulation. Multivariate linear regression analysis using the stepwise backward-selection method was also performed to identify the independent predictors of SAF (measured as a continuous variable) after adjusting for the significant univariate variables. Unadjusted P-values less than 0.05 were considered significant.

## Results

### Patient´s characteristics

**Fig 1:**
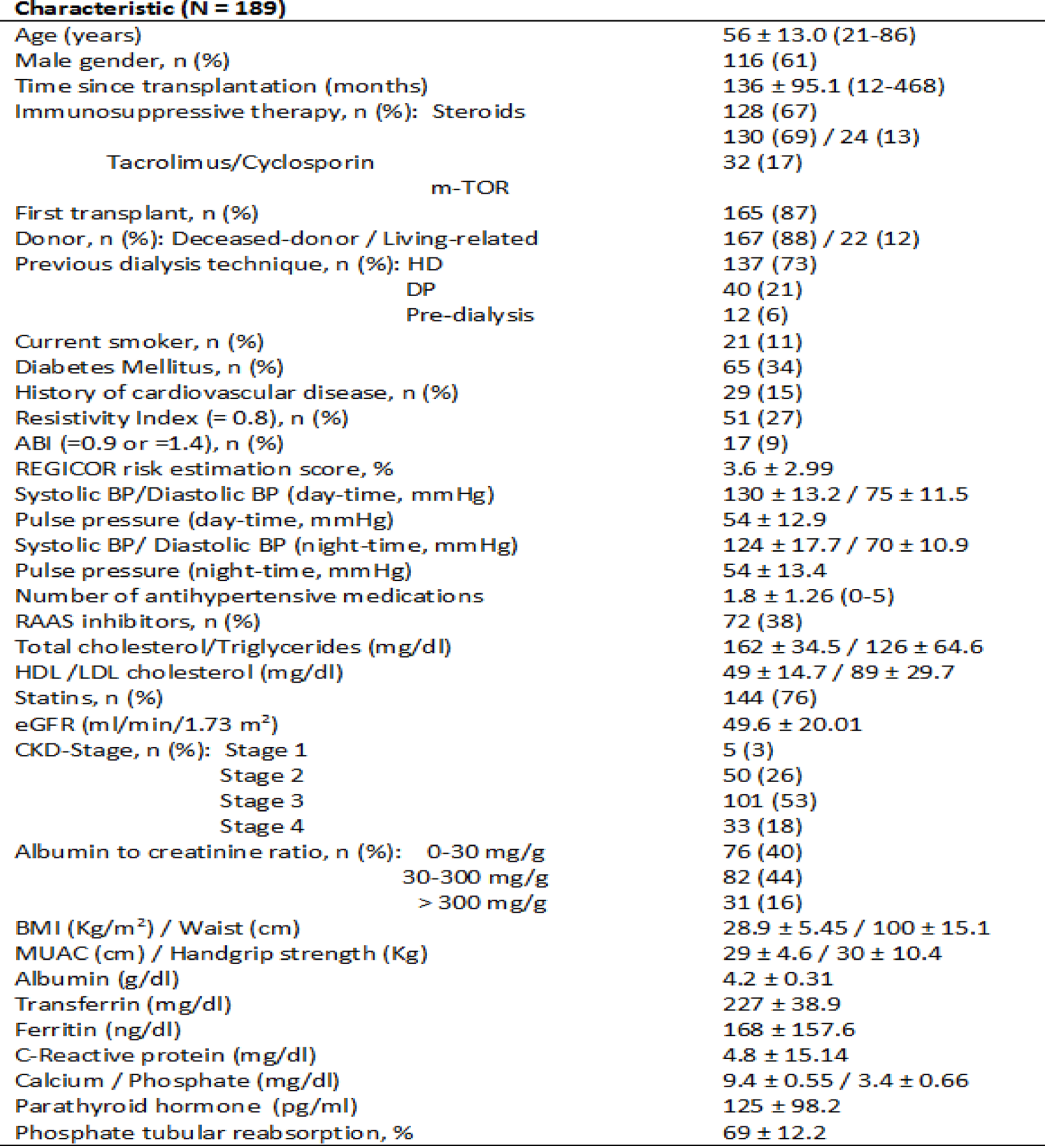
Patient’s characteristics. Data is expressed as means ± standard deviation. Range is in brackets when appropriate. HD = Hemodialysis, PD = Peritoneal Dialysis, ABI = Ankle-Brachial Index, BP = Blood Pressure, RAAS = Renin-Angiotensin-Aldosterone System, eGFR = Estimated Glomerular Filtration Rate, CKD = Chronic Kidney Disease, BMI = Body Mass Index, MUAC = Mid-upper arm circumference.

Results, for the total 189 enrolled patients (116 males and 73 females), are presented in Fig 1. Mean age of the group was 56 ± 13.0 years. Time from transplantation ranged from 12 to 468 (median 118) months. Diabetes was present in 34% of cases (65 patients) whereas diabetic nephropathy accounted only in 17 cases (9%). Major vascular events, namely coronary heart disease, cerebrovascular and peripheral artery disease were present in 29 patients (15%). Actual immunosuppressive medications included steroids (128 patients), tacrolimus (130 patients), cyclosporine A (24 patients), m-TOR inhibitors (32 patients) and mycophenolate (158 patients). 22 patients (12%) had a living related donor. Patients were on a mean of 1.8 ± 1.26 antihypertensive medications. 72 patients were receiving renin-angiotensin-aldosterone system (RAAS) inhibitors and 76% were on statins. 21 patients (11%) were currently smokers. Pretransplant dialysis technique was hemodialysis (137 patients) and peritoneal dialysis (40 patients).

Graft function estimated by the CKD-EPI was 49.6 ± 20.01 ml/min/1.73 m^2^. According to CKD stages (KDOQI classification), 5 patients (3%) were on stage 1, 50 (26%) on stage 2, 101 (53%) on stage 3 and 33 (18%) on stage 4. Whereas microalbuminuria was present in 44% of cases, 16% showed overt proteinuria. Nutritional parameters, namely mean BMI and handgrip strength, were 28.9 ± 5.45 kg/m^2^ and 30 ± 10.4 Kg respectively.

### Correlations of skin AF with demographic, clinical and biological parameters

**Fig 2:**
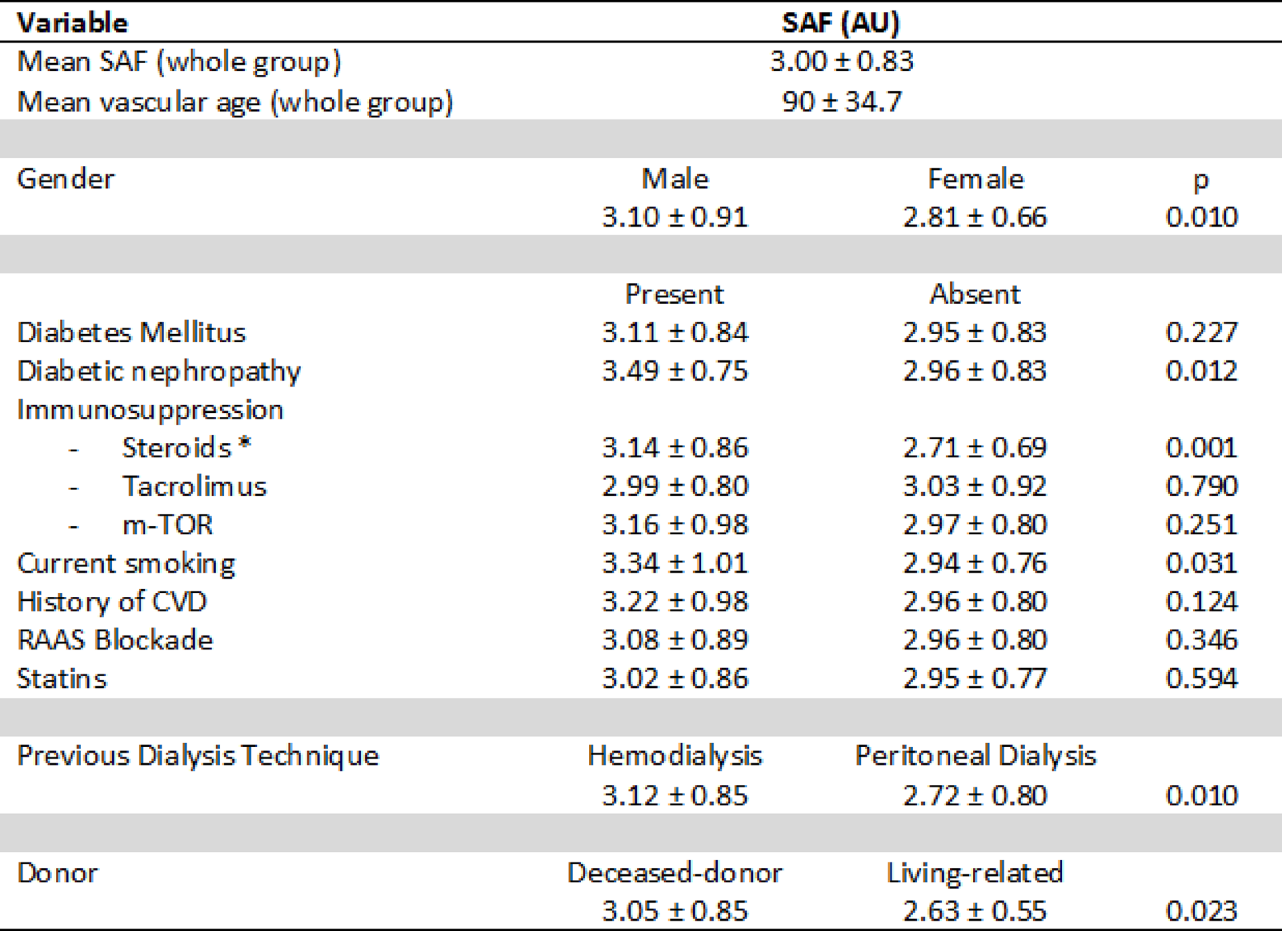
SAF values in selected categorical variables. Data is expressed as means ± standard deviation. SAF = Skin Autofluorescence, AU = Arbitrary Units, CVD = Cardiovascular Disease, RAAS = Renin-Angiotensin-Aldosterone System. *Steroids refers as steroid use at the time of SAF determination (present or absent).

Mean skin autofluorescence was 3.00 ± 0.83 (1.5–6.4) AU. Estimated vascular age for the whole group was 90 ± 34.7 years (>30 years older than chronological age). Comparison of potentially relevant categorical variables (gender, diabetes type of previous dialysis, donor, immunosuppression, etc.) are showed in Fig 2. SAF levels were significantly higher in men (3.10 ± 0.91 vs 2.81 ± 0.66 AU) and in those with diabetic nephropathy. Interestingly, RT recipients with a history of peritoneal dialysis (PD) showed SAF values significantly lower than those with a history of hemodialysis (HD). Though not specifically analyzed, a shorter dialysis vintage for PD, age or a better-preserved residual renal function prior to transplant in PD patients might be responsible of this finding. Likewise, transplants from a living-related donor also showed less SAF levels (2.63 ± 0.55 vs 3.05 ± 0.85 AU). Regarding immunosuppression regimen, patients currently on steroids had higher SAF intensity than those without (3.14 ± 0.86 vs 2.71 ± 0.69). Subsequently, vascular age was significantly higher among steroid treated patients (96 ± 36.0 years vs 78 ± 28.6 years). This difference was not attributable either to diabetes or chronological age since patients with and without steroids had comparable ages (57 ± 11.8 vs 55 ± 15.2 years, respectively) and a lower diabetes frequency (31 vs 42%, respectively). No other differences were found regarding immunosuppression therapy, time since transplantation, statin or RAAS inhibitors use. Despite active smokers were younger than non-smokers (45 ± 15.1 vs 58 ± 12.6 years), they statistically showed higher SAF-values than non-smokers.

**Fig 3:**
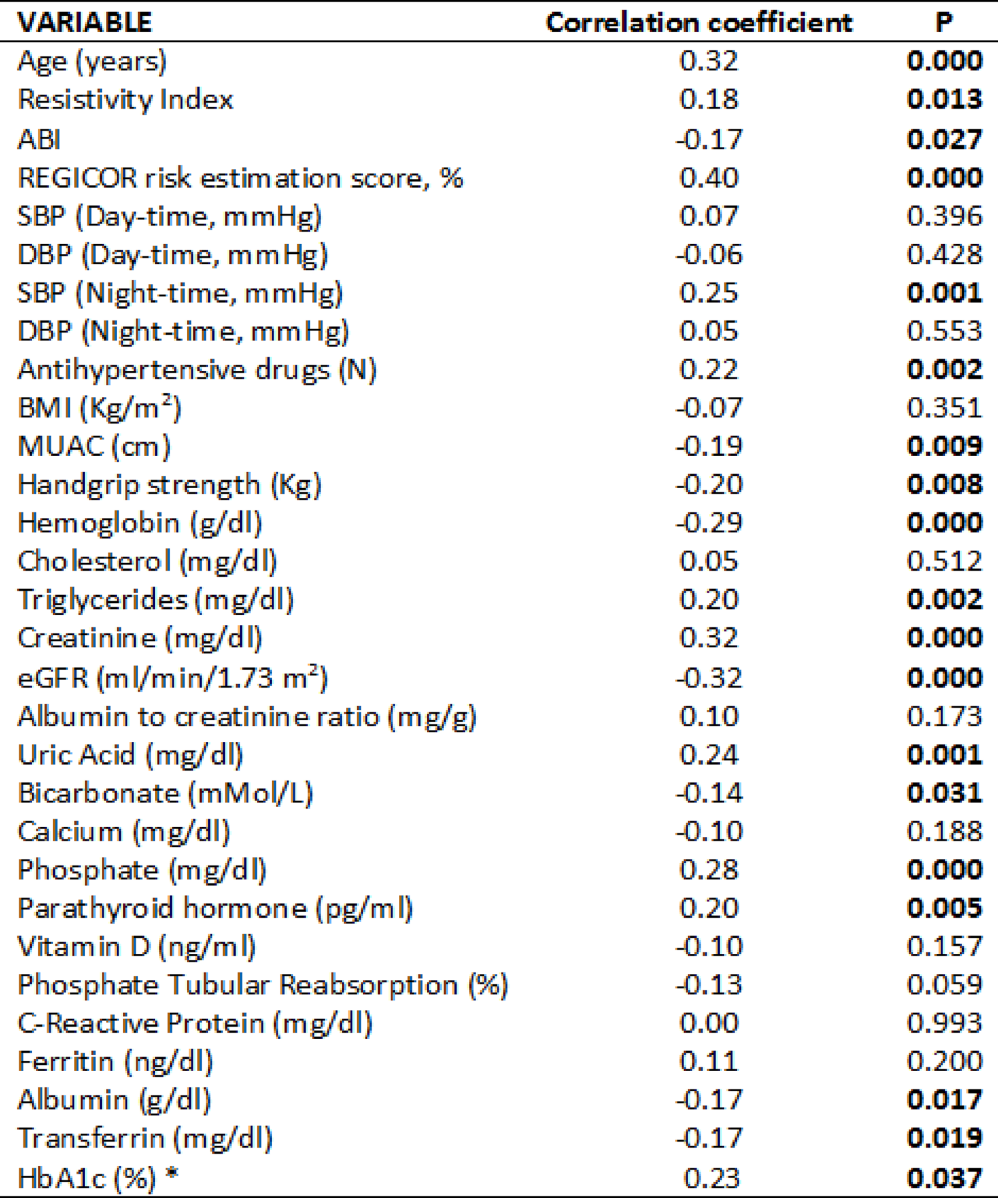
Univariable correlations between SAF and continuous variables. ABI = Ankle-Brachial Index, SBP = Systolic Blood Pressure, DBP = Diastolic Blood Pressure, BMI = Body Mass Index, MUAC = Mid-upper arm circumference, eGFR = Estimated Glomerular Filtration Rate, HbA1c = Glycated hemoglobin, *Diabetic patients only, Significant variables are shown in negrita.

Univariate correlates of SAF are displayed in Fig 3. As expected, SAF showed a positive correlation with age (r = 0.32). SAF intensity was also positively associated with nighttime systolic blood pressure, the number of antihypertensive drugs, triglycerides, uric acid, PTH and phosphate concentration. Phosphate tubular reabsorption rate showed a negative correlation (r = -0.13, p = 0.05). Renal function (eGFR), bicarbonate and hemoglobin were also negatively correlated with SAF. Nutritional status-related variables, including serum albumin, transferrin, MUAC and handgrip strength also showed a negative correlation with SAF. No significant association was observed with BMI, waist, reactive protein C, ferritin, albuminuria, vitamin D, calcium and total cholesterol or its fractions. In diabetics, glycated hemoglobin (%) was positively correlated with SAF (r 0.23; p = 0.037).

**Fig 4:**
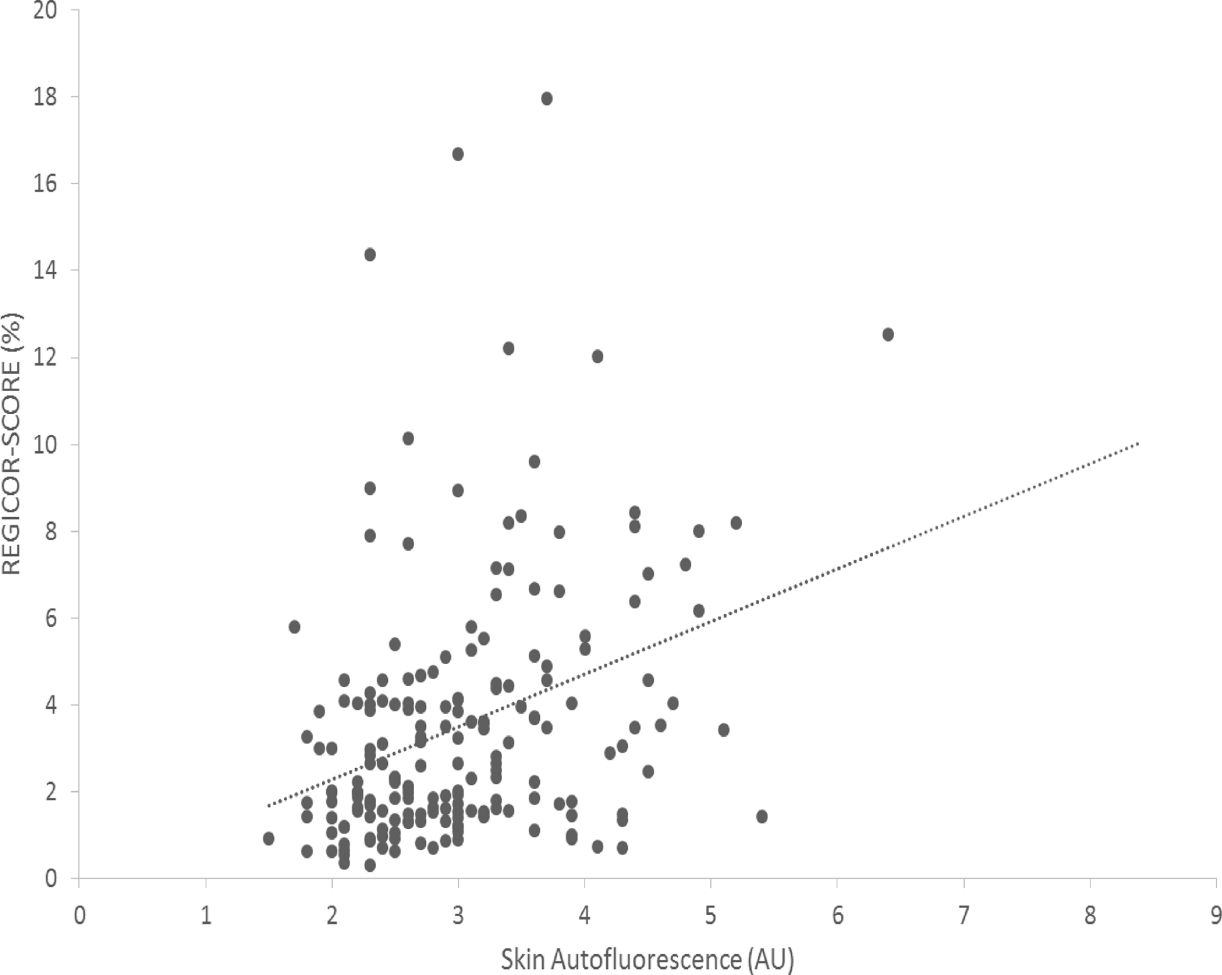
Correlation between SAF and the REGICOR-risk score. r = 0.35, p < 0.001; AU = Arbitrary Units.

SAF was associated with atherosclerosis damage estimated by ABI (r = -0.17) and intragraft resistivity index (r = 0.18). Of note, as shown in Fig 2, SAF values were not statistically associated with a history of cardiovascular event (probably due to the reduced number of patients with this condition in the series). Nonetheless, application of the REGICOR scale showed a very close correlation with SAF (Fig 4).

### Linear regression analysis

**Fig 5:**
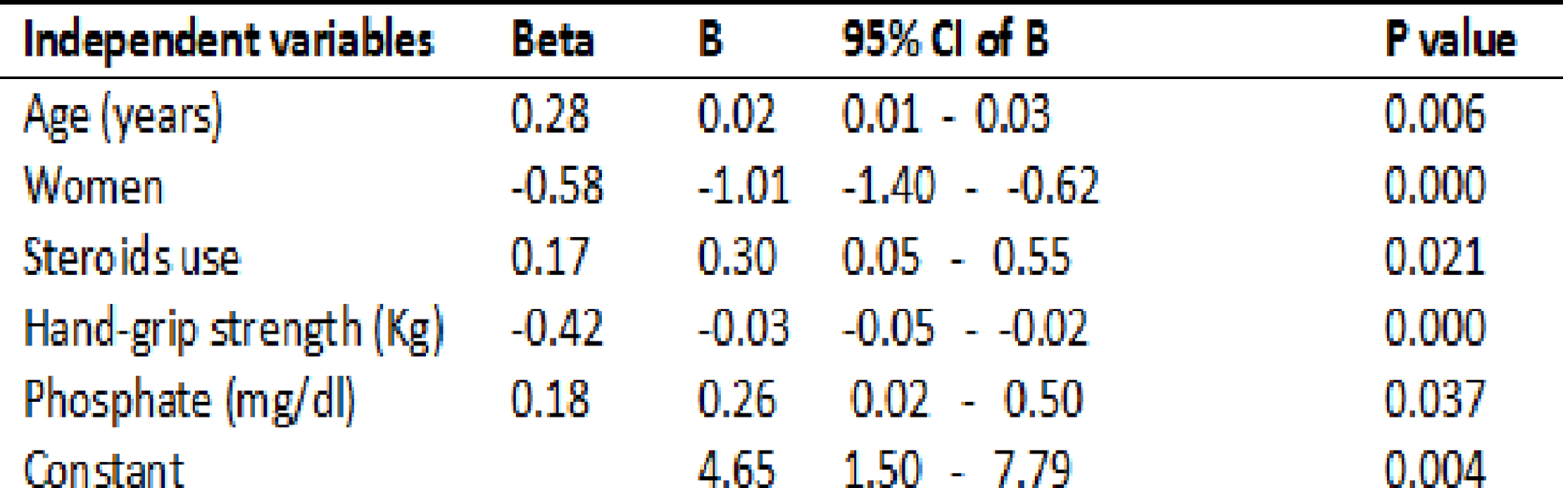
Stepwise multivariable linear regression model (SAF = dependent variable). R^2^ = 0.535, p < 0.001. Variables stepwise excluded: Diabetes Mellitus, renal function (eGFR), uric acid, bicarbonate, systolic blood pressure (night-time), number of antihypertensives, hemoglobin, albumin, parathyroid hormone, triglycerides, transferrin, REGICOR risk score, Resistivity index and ABI.

Stepwise linear regression analysis was performed to determine the parameters that independently associated to SAF. In a final model which explained 53% of the variance of skin-AF, age, male gender, steroids use, serum phosphate and handgrip strength emerged as independent contributors, as shown in Fig 5. Renal function (CKD-EPI), uric acid, systolic blood pressure (night-time), number of antihypertensives, hemoglobin, albumin, bicarbonate, parathormone, triglycerides and transferrin did not significantly influence SAF in the model. Addition of REGICOR risk score, Resistivity index, ABI, replacement of diabetes with diabetic nephropathy, type of donor or previous dialysis technique (HD or PD) in the model did not change the results.

## Discussion

SAF is a marker of AGEs accumulation and predictor of CVD mortality in aging, diabetes mellitus and CKD [11–16]. We performed a cross-sectional study to find out clinical determinants for SAF among renal transplant patients. SAF levels in our study were comparable with previous reports pinpointing RT as an incomplete solution for AGE accumulation in uremia [21, 22]. Several vascular and transplant-specific factors may explain this AGEs excess after RT and are also strengthened by our results. AGEs are considered to affect the biomechanical properties of matrix proteins, thereby contributing to the physiological process of chronological aging [7, 26]. Using a validated equation based on reference SAF values [23], we observed a marked increase in the vascular age of our patients (more than 30 years higher than chronological age). This disparity has also been documented in a recent research of Sanchez et al within CKD population with subclinical atherosclerosis [27].

We also found an association between SAF intensity and male sex that has not been consistently seen in previous studies also carried out after RT [21, 22]. However, sex-differences in SAF values may not be fully attributed to sex per se but to underlying comorbidities and conditions like diabetes, CVD, CKD, smoking and dietetic habits among others, that may prevail in one sex over the other [11, 28]. Ovarian dysfunction common in uremia and the decrease in skin collagen after menopause may also account for these diverse associations [29–30].

Glycation adducts in tobacco and smoke can form cross-links with proteins [31]. Despite the low percentage of smokers included (11%) and their younger age, our results support the harmful association of smoking in AGEs accumulation after RT. In contrast, our data did not observe other previously reported conditions linked with a favorable AGEs´ balance like RAAS blockade or statins [32]. However, the cross-sectional design of our investigation is not suitable to study the potential anti-AGE benefit of these treatments after renal transplantation.

As expected, we have found a relation with diabetic nephropathy but it did not stand multivariable adjustment. Moreover, postransplant diabetes was also not statistically associated with SAF. Diabetes might be a sufficient condition but it is not essential for AGE cardiovascular damage [5]. We speculate that other conditions beyond diabetes (such as chronic inflammation and nutritional status) may interact with the unfavorable post-transplant metabolic *milieu* of dyslipemia, insulin resistance and reduced antioxidant defenses. Collectively, this may enhance both lipid and protein glycosylation contributing to elevated SAF values [20, 33, 34]. Moreover, in the presence of diabetes, our results also show a positive correlation between SAF and HbA1c.

Interestingly, a novel finding in our study was the independent association between use of steroids and SAF intensity, something that would need confirmation in subsequent studies. The wide range of post-transplant period (12–468 months) that may include different immunosuppressive protocols (including steroid use and withdrawal strategies) and the cross-sectional design of the study (comparing patients with and without steroids at that moment), may interfere with this observation. Moreover, as only patients from a single center were enrolled, these results are not to be generalized without further considerations.

It has been proposed that in the presence of certain risk factors, circulating AGEs may deposit in blood vessels including graft microvasculature and tubular cells, contributing to renal function decline [12,35]. In chronic allograft nephropathy, Raj et al [34], showed that AGEs might even increase independently of renal function itself reinforcing this vicious cycle. In our study, renal function but not proteinuria, was significantly correlated with SAF. However, multivariable analysis of data failed to support graft function as an independent marker of SAF. Because age and sex play an important role in the calculation of eGFR, the observed association perhaps represents a residual confounding [36]. Serum AGEs are linked to high turnover proteins that may rapidly improve with a healthy graft [19, 33]. In the long run after transplantation, formation, accumulation, breakdown and clearance of tissue AGEs on long-stable tissue proteins is a slow process that reflects accumulative metabolic stress [37]. This idea is also supported by our study by the finding that PD and living donor recipients, with a shorter dialysis vintage or a better preserved residual renal function prior to transplantation, had lower levels of SAF in our analysis. Crowley and colleagues, reported a relationship between SAF and time since transplantation in a previous work [38]. Unexpectedly, our results failed to prove this relation. The wide range of post-transplant period (12–468 months) and the exclusion of patients with less than one year after transplant (who could dramatically improve relatively soon in the short-term after RT) might explain this result.

In contrast to other studies [21,22], we did not find an association between SAF and inflammation (estimated by either ferritin or C-reactive protein). The wide range of post-transplant observation period and the exclusion criteria applied in our study may explain this result. On the other hand, an interesting and previously unknown observation from of our study is the consistent association between SAF intensity both nutritional biochemical markers (i.e. albumin, transferrin) and muscle-related parameters (MUAC and handgrip strength). Sarcopenia has been recently linked with AGEs accumulation and SAF in Japanese population [39]. Furthermore, oxidant stress, inflammation and malnutrition characterize a novel unifying model for CVD risk in uremia [40]. Protein energy wasting (PEW) is an independent factor associated with morbi-mortality in both CKD and renal transplantation with a major impact at dialysis stage [41]. Anemia, another associate of SAF in our study, has been also significantly linked with PEW in renal transplants [42]. Though RT may share similar scores for malnutrition-inflammation with eGFR-paired CKD patients [41], not all these correlates have been studied in depth [43].

Uremic mineral-metabolism disturbances (CKD-MBD) are largely restored after successful renal transplantation. For instance, fibroblast growth factor (FGF-23) returns to normal values one year after RT though hyperparatiroidism and relatively low phosphate levels may persist in the long-term [19]. However, even average values of serum phosphate (> 3.5 mg/dL), are predictors of all-cause mortality after 4 years of RT independently of allograft function [44]. We found an interesting correlation between serum phosphate, phosphate excretion, parathyroid hormone and SAF. Unfortunately we did not analyze other components of the CKD-MBD axis like klotho or FGF-23 levels. However, if phosphate control or AGE restriction may influence RT outcome requires prospective studies.

Beyond phosphate influence, AGEs deposit in the vessel wall and atherosclerotic plaques have been postulated to contribute to the progression of CVD in diabetes or CKD and to associate with the risk of mortality [12, 45, 46]. Limited by the reduced number of patients with cardiovascular events in our series, we failed to find a significant association with skin AF and major CVD. Instead, we used surrogate markers for subclinical atherosclerosis like ABI, RI and nocturnal BP (a marker of arterial stiffness) and observed univariate associations. Our findings might be also in agreement with previous reports that argue in favor of graft RI as a correlate of recipient´s hemodynamic and vascular characteristics rather than with graft function itself [47, 48]. Finally, our results show an interesting correlation between SAF and the REGICOR-calibrated Framingham´s cardiac risk equation. The multimorbidity nature of our patients may explain this association, while contrast with a previous report observing no-link between cardiovascular risk SCORE and SAF in non-CKD, non-diabetic participants [49].

## Conclusions

As hypothesized, skin AF may represent a clinically useful, non-invasive method, to assess cardiovascular risk in RT. Limitations of our analysis are, however, its cross-sectional nature that precludes any causation in the associations observed, and the lack of circulating or direct tissue AGEs assessment. Besides age and male gender, our results indicate that serum phosphate, steroid use and nutritional status are the major determinants of SAF after long-term RT. If AGE deposition is the corner stone linking PEW, glucocorticoids and CVD after RT as well as their role as a new therapeutic target requires further prospective investigations.
